# YAP as a therapeutic target to reverse trastuzumab resistance

**DOI:** 10.1007/s10120-025-01630-w

**Published:** 2025-06-20

**Authors:** Ah-Rong Nam, Kyoung-Seok Oh, Ju-Hee Bang, Yoojin Jeong, Sea Young Choo, Hyo Jung Kim, Su In Lee, Jae-Min Kim, Jeesun Yoon, Tae-Yong Kim, Do-Youn Oh

**Affiliations:** 1 Cancer Research Institute, Seoul National University College of Medicine, Seoul, 03080 Korea; 2 Integrated Major in Innovative Medical Science, Seoul National University Graduate School, Seoul, 03080 Korea; 3 Department of Internal Medicine, Seoul National University Hospital, Seoul, 03080 Korea

**Keywords:** Trastuzumab resistance, HER2, YAP, Hippo signaling pathway, Verteporfin

## Abstract

**Background:**

Trastuzumab resistance in HER2-positive cancers remains a significant clinical challenge with limited therapeutic options. Although the tumor-promoting role of the Yes-associated protein (YAP) pathway is well established, its role in trastuzumab resistance remains unclear.

**Methods:**

We established four trastuzumab-resistant (HR) cell lines (NCI-N87HR, SNU216HR, SNU2670HR, and SNU2773HR) from HER2-positive gastric cancer and biliary tract cancer cell lines. YAP pathway activation was assessed using Phospho-RTK arrays, bulk RNA-Seq, and immunofluorescence. Antitumor effects of YAP targeting were evaluated with MTT assays, cell-cycle analysis, migration assays, RT-qPCR, ELISA, and xenograft models of SNU-2773 and SNU-2773HR cells. Immune modulation by YAP was studied through co-culture experiments with human PBMCs and cancer cells, followed by flow cytometry analysis of immune markers.

**Results:**

Upregulation and activation of the YAP/TAZ pathway were observed in HR cells, indicated by elevated ROR2 levels and nuclear translocation of YAP. This activation, driven by YAP/TEAD-dependent Wnt5a expression, suggests a positive-feedback mechanism that amplifies YAP activity. Elevated YAP and TEAD levels were observed in patient tumor tissues during disease progression following HER2-targeted therapies. Targeting YAP disrupted its oncogenic effects and restored sensitivity to trastuzumab, increased activation of CD4^+^ and CD8^+^ T cells in PBMCs, likely via PD-L1 downregulation and enhanced immunogenic cell death. Verteporfin, a YAP-TEAD inhibitor, effectively reduced tumor growth and increased apoptosis in mouse models bearing HR tumors.

**Conclusions:**

Targeting the ROR2-YAP/TEAD axis presents a promising therapeutic approach to overcome trastuzumab resistance in HER2-positive cancers, offering a potential strategy for enhancing treatment efficacy and improving clinical outcomes.

**Supplementary Information:**

The online version contains supplementary material available at 10.1007/s10120-025-01630-w.

## Background

Aberrant human epidermal growth factor receptor 2 (HER2) signaling, due to genetic alterations or overexpression via myriad biological mechanisms, facilitates the neoplastic conversion of premalignant cells and tumor progression [1]. Strategy of targeting HER2 in combination with chemotherapy had been the standard-of-care for patients detected with HER2-positive (IHC3 + or IHC2 + and ISH +) breast cancer (BC) and gastric cancer (GC) over decades by demonstrating survival benefit in the patient subset [2–4]. Trastuzumab, an anti-HER2 monoclonal antibody, was the first approved therapeutic agent among multiple classes of anti-HER2 agents and remains the mainstay in the treatment of HER2-positive cancers, along with HER2-directed antibody–drug conjugates (ADCs) and bispecific antibodies [5, 6]. The advent of ADCs has opened new horizons in the treatment landscape of HER2-positive tumors, with enhanced antitumor effects compared to the conventional anti-HER2 therapies [7–11]. Moreover, the HER2-directed ADC trastuzumab–deruxtecan (T-DXd) showed potent antitumor activity against HER2-low (IHC2 + /ISH- or IHC1 +) BC [12]. However, resistance to trastuzumab remains a critical issue as even the most effective drugs ultimately encounter resistance. Although many new HER2-ADCs are emerging and may eventually replace trastuzumab in certain settings, trastuzumab remains the backbone of many therapies [13]. Therefore, understanding the mechanisms underlying the acquired resistance to trastuzumab is essential for developing better therapeutic strategies. Our study focused on elucidating the resistance mechanisms of trastuzumab, aiming to identify vulnerabilities that can be exploited to improve treatment outcomes.

Extensive studies have dissected the accumulating types of molecular events that are responsible for resistance to trastuzumab, which could generally be categorized into (1) alterations in HER2 at a genetic level (HER2 activating mutations, splice variants, and truncations) or at a molecular level (nuclear localization), (2) alternative activation of other oncogenic pathways, (3) metabolic reprogramming, and 4) evasion from antitumor immunity. Despite the advent of potential therapeutic strategies based on these known mechanisms, so far only few have been successfully translated into the clinical setting [14–16].

Yes-associated protein (YAP) signaling, which is tightly regulated by multiple extracellular factors such as an evolutionary conserved Hippo pathway and non-canonical Wnt/β-catenin pathway, is aberrantly expressed and activated in various types of tumors [17]. Tumor-promoting roles by regulating cell proliferation, apoptosis, metastasis, and cancer stem-cell-like properties make the YAP pathway a promising therapeutic target for multiple types of solid tumors [18].

The involvement of the YAP/TEAD axis in trastuzumab and T-DM1 resistance has been identified through multiomic analyses of HER2-positive cancer cells and tumor samples from patients resistant to anti-HER2 regimens, highlighting the enhanced expression and activation of this pathway [19–22]. However, the precise molecular mechanisms driving YAP dysregulation in trastuzumab resistance and the therapeutic implications of the Hippo pathway effectors remain unclear. Although the HER4–YAP1 axis has been linked to epithelial–mesenchymal transition in trastuzumab-resistant (HR) GCs [23], a comprehensive understanding of the role of YAP in HER2-positive tumors is not available.

Based on our finding of increased ROR2 expression in HR cell lines, we investigated the therapeutic role of the YAP pathway in trastuzumab resistance in HER2-positive cancer cells.

## Methods

### Cell lines & reagents

Four human cancer cell lines with HER2 amplification were used in this study. NCI-N87 and SNU-216 cells (GC cell lines) were purchased from the Korean Cell Line Bank (Seoul, Korea). Patient-derived biliary tract cancer (BTC) cell lines with HER2 amplification (SNU-2670 and SNU-2773) were established as previously described [24]. HR cell lines derived from the above-mentioned cell lines (SNU-2670HR and SNU-2773HR) were generated as previously described [25]. Cell lines were maintained in RPMI medium (Welgene Inc. Gyeongsan, Korea), 10% FBS, and 10 µg/ml gentamycin at 37 °C with 5% CO_2_. Verteporfin (S1786), CA3 (CIL56; #S8661), and Ozuriftamab (#S8758) were purchased from Selleck Chemicals (Houston, TX, USA). Recombinant Human/Mouse Wnt-5a Protein (#645-WN-010) and cycloheximide (#C7698) were purchased from R&D Systems (Minneapolis, MN, USA) and Sigma-Aldrich (St. Louis, MO, USA), respectively.

### Immunoblotting

Immunoblot assays were performed as previously described [26]. Briefly, protein samples were prepared from cell lysates using SDS sample loading gel electrophoresis (SDS-PAGE). Separated proteins were transferred onto a nitrocellulose blotting membrane and blocked with 1% nonfat milk/bovine serum albumin (BSA)-supplemented Tris-buffered saline and Tween 20 buffer. Primary and secondary antibody incubations were performed, followed by chemiluminescence detection. Information of antibodies used in this study are as follows; Santa Cruz Biotechnology (Dallas, TX, USA); anti-GAPDH (#sc-25778), anti-Cyclin E (#sc-247), Cell Signaling Technology (Danvers, MA, USA); anti-ROR1 (#cst-4102), anti-ROR2 (#cst-4105), anti-Phospho-YAP (Ser127) (#cst-13008), anti-YAP (#cst-14074), anti-Wnt5a (#cst-2392), anti-TAZ (#cst-83669), anti-Pan-TEAD (#cst-13295), anti-Bcl-xL (#cst-2764), anti-PD-L1 (#cst-13684), anti-Phospho-Stat3 (#cst-9131), anti-IRF1 (#cst-8478), anti-Phospho-Stat1 (Tyr701) (#cst-9167), anti-CTGF (#cst-86641), anti-Cyclin D1 (#cst-2978), anti-p27 (#cst-3686), anti-PARP (#cst-9532), anti-XIAP (#cst-14334), anti-Bax (#cst-14796), anti-β-Actin (#cst-3700), Invitrogen (Waltham, MA, USA); anti-CD63 (#MA1-19281), Abcam (Cambridge, UK); anti-Lamin B1 (#ab16048).

### Immunofluorescence

Cells seeded in glass-bottom culture dishes were fixed with 4% paraformaldehyde (Biosesang, Yongin, Korea) and permeabilized with 0.5% Triton X-100 (Sigma-Aldrich, #X100). It was then blocked with 2% BSA/PBS, followed by incubation with primary antibody, anti-YAP antibody (Cell Signaling Technology, #cst-14074), and incubated overnight. After secondary antibody incubation with Alexa Fluor 488 goat anti-rabbit IgG (Invitrogen, #A-11008), nuclei were counterstained with DAPI and subjected to fixation with an antifade mounting solution (Vector Labs, Newark, CA, USA, #H-1000-10). Images were acquired using a STELLARIS 5 confocal microscope (Leica Microsystems).

### Cell viability assay

Cells were incubated for a predetermined period in 96-well plates and treated with 3-(4,5-dimethylthiazol-2yl)-2,5-diphenyltetrazolium bromide (MTT) solution (Tokyo Chemical Industry Co., Ltd., Tokyo, Japan; #D0801) for 4 h. Post-incubation, to determine cell viability, MTT formazan was dissolved in DMSO and colorimetric absorbance was measured at 540 nm using a Multiskan GO spectrophotometer (Thermo Fisher Scientific, Waltham, MA, USA).

### The siRNA transfection

The cells were transfected with 50 nM siRNA targeting Wnt5a or YAP1 (Genolution, Seoul, Korea) using Lipofectamine 2000 (Thermo Fisher Scientific) in a serum-free medium for 6 h. The medium was then replaced with 10% FBS culture medium and the cells were incubated for 24 h with siYAP1. For siWnt5a, this process was repeated, resulting in a total incubation time of 48 h. Transfected cells were harvested and reseeded for subsequent experiments. Sequence information on siRNAs are as follows; negative control: sense 5′-AAU UCU CCG AAC GUG UCA CGU UU-3′, anti-sense 5′- ACG UGA CAC GUU CGG AGA AUU UU-3’, siYAP#1: sense 5′-GAU GGA UAC AGG UGA UAC UUU-3′, anti-sense 5′-AGU AUC ACC UGU AUC CAU CUU-3′, siYAP#2: sense 5′-GUA UUG CUG ACC UCU UUC AUU-3′, anti-sense 5′-UGA AAG AGG UCA GCA AUA CUU-3’, siWnt5a#1: sense 5’-GAA ACU GUG CCA CUU GUA UUU-3′, anti-sense 5′-AUA CAA GUG GCA CAG UUU CUU-3′, and siWnt5a#2: sense 5′-CAA AGA AUG CCA GUA UCA AUU-3′, anti-sense 5′-UUG AUA CUG GCA UUC UUU GUU-3′.

### RT-qPCR

Total RNA was separated using TRIzol (Thermo Fisher Scientific, #15,596,018) and chloroform (Sigma-Aldrich, #288,306), and precipitated with isopropanol. Using purified RNA, cDNA was synthesized using the ImProm-II™ Reverse Transcription System (Promega, Madison, WI, USA, #A3800) following the manufacturer’s instruction. Real-time PCR was performed using TOPreal SYBR Green qPCR PreMIX (Enzynomics, Korea, #RT500M) on a QuantStudio 3 Real-Time PCR Instrument (Applied Biosystems, Waltham, MA, USA). Primers used for the RT-qPCR are as follows; YAP: sense 5′-GGC TGA AAC AGC AAG AAC TG-3′, anti-sense 5′-GAA GAC ACT GGA TTT TGA GTC-3′; AREG: sense 5′-GAG CCG ACT ATG ACT ACT CAG A-3′, anti-sense 5’-TCA CTT TCC GTC TTG TTT TGG G-3′; CTGF: sense 5′-CAG CAT GGA CGT TCG TCT G-3′, anti-sense 5′- AAC CAC GGT TTG GTC CTT GG-3′; CYR61: sense 5′-GGT CAA AGT TAC CGG GCA GT-3′, anti-sense 5′-GGA GGC ATC GAA TCC CAG C-3′.

### Cell cycle analysis

Cells collected by trypsinization were fixed with 70% ethanol for at least 48 h. The cells were subjected to flow cytometric analysis of cell-cycle distribution following the sequential addition of RNase A and propidium iodide using the BD FACSCanto II system (BD Biosciences, Franklin Lakes, NJ, USA).

### Wound-healing assay

Confluent cell monolayers seeded in 6-well plate were gently scratched using a 200 µl pipet tip and treated with vehicle (PBS) or verteporfin After 24 or 48 h of incubation, cell migration into the scratched area was observed under a CKX41 inverted microscope (Olympus, Tokyo, Japan). The extent of wound closure was determined with 10 measurements in each experiment using the ImageJ software.

### ELISA

The extracellular release of CCL5, CXCL10, and HMGB1 was measured using the Human CCL5/RANTES Quantikine ELISA Kit (R&D Systems, #DRN00B), Human CXCL10/IP-10 Quantikine ELISA Kit (R&D Systems, #DIP100), and Human HMGB1 ELISA Kit (Assay Genie, #HUFI00660). All procedures were performed according to the manufacturer’s instructions.

### Flow cytometry

Cells were collected by trypsinization, and 1 × 10^6^ cells were analyzed using the BD FACSCanto II system (BD Biosciences) after 30 min of antibody incubation in cell staining buffer (BioLegend, #420201). The antibodies used were as follows: APC anti-human CD274 (B7-H1, PD-L1) antibody (BioLegend, #329708) and Calreticulin antibody (1G6A7) [FITC] (Novus Biologicals, #NBP1-47518F).

### Caspase-3 activity

Caspase-3 activity was assessed using the EnzChek^®^ Caspase-3 Assay Kit #1 (Thermo Fisher Scientific, #E-13183) according to the manufacturer’s instructions. Briefly, cancer cells pre-treated with verteporfin for 48 h were co-cultured with PBMCs for additional 72 h. After co-culture, cells were harvested and lysed with 1 × Cell Lysis Buffer. Lysates were centrifuged to remove cellular debris, and 50 µL of the supernatant from each sample was transferred to a 96-well plate. Subsequently, 50 µL of a 2 × substrate solution containing Z-DEVD–AMC was added to each well. Following incubation for 30 min at room temperature in the dark, fluorescence was measured using a microplate reader.

### Phospho-RTK array

The Proteome Profiler Human Phospho-RTK Array Kit (R&D Systems, #ARY001B) was used for the phospho-RTK array. Reagents and samples were prepared according to the manufacturer’s instructions. Briefly, arrays were incubated for 1 h at room temperature. Cell lysates were prepared and added to the arrays, followed by overnight incubation at 4 °C. The arrays were then washed three times with wash buffer for 10 min each. The anti-phospho-tyrosine-HRP detection antibody was added and it was incubated for 2 h. Chemiluminescence detection was performed by adding the Chemi Reagent Mix and exposing the membranes to an X-ray film.

### Bulk RNA-Seq

Total RNA was extracted using the TRIzol reagent and verified using a TapeStation RNA Screentape (Agilent, #5067-5576). Samples with an RNA integrity number (RIN) > 7.0 were used for library construction using the TruSeq Stranded mRNA Sample Prep Kit (Illumina, #RS-122-2101). Libraries were quantified using KAPA Library Quantification kits and assessed using a TapeStation D1000 ScreenTape (Agilent, #5067-5582) before sequencing on an Illumina NovaSeq platform. Raw reads were trimmed using Trimmomatic 0.38 and aligned to the Homo sapiens reference genome (GRCh38) using HISAT v2.1.0. Transcripts were assembled and read counts per gene were calculated using StringTie v2.1.3b. Differentially expressed genes (DEGs) were identified with DESeq2 (|fold change|≥ 2, raw *p* < 0.05). Hierarchical clustering was performed using complete linkage and Euclidean distances. Functional and pathway analyses were conducted using gProfiler.

### Exosomal PD-L1 analysis

Total exosomes were isolated using the Total Exosome Isolation Reagent (Invitrogen, #4478359) following the manufacturer’s instructions. Briefly, cell-free cell culture media were mixed with the reagent at a ratio of 2:1 and incubated for 24 h at 4 °C. After centrifugation at 10,000 × *g* for 1 h at 4 °C, pellet containing exosomes were resuspended in PBS for subsequent immunoblotting.

### PBMC co-culture experiments

Peripheral blood mononuclear cells (PBMCs) were isolated from peripheral blood using Ficoll gradient centrifugation (Ficoll–Paque Plus, Cytiva, #17-1440-03) and stored in the deep freezer until use. Cancer cells pre-treated with verteporfin for 48 h were harvested and reseeded in a 100 mm culture dish. After 24 h, PBMCs were thawed and co-cultured with cancer cells at a ratio of 5:1 (effector to target cells) for additional 72 h with and without the CD3/CD28 T Cell Activator (#10991, STEMCELL Technologies). Flow cytometry was used to determine the proportion of T cells and PD-1 expression in CD8^+^ T cells. The antibodies used are as follows: CD3 monoclonal antibody (UCHT1), APC (Invitrogen, #17-0038-42), CD4 monoclonal antibody (RPA-T4), PE (Invitrogen, #12–0049-42), CD8a monoclonal antibody (RPA-T8), FITC (Invitrogen, #11-0088-42), CD279 (PD-1) monoclonal antibody (MIH4), PE (Invitrogen, #11-9969-42), Pacific Blue™ anti-human/mouse granzyme B recombinant antibody (Biolegend, #372,218), APC/Cyanine7 anti-human CD14 antibody (BioLegend, #325620), PE anti-human CD11c antibody (BioLegend, #337206), CD80 (B7-1) antibody, FITC (eBioscience™, Invitrogen, #11-0809-42), and APC-anti-human HLA-DR antibody (Biolegend, #307610).

### Mouse tumor xenograft

Animal experiments were conducted at the Institute for Experimental Animals, College of Medicine, Seoul National University (Seoul, Korea), following institutional guidelines, with prior approval from the Institutional Animal Care and Use Committee (No. SNU-211223-1-1). A xenograft tumor model was established administering a subcutaneous injection of 1 × 10^7^ SNU-2773 or SNU-2773HR cells into the right flank of a 4-week-old female BALB/c nude mice (Orient Bio Inc., Seongnam, Korea). When the tumors reached approximately 200 mm^3^, the mice were randomly divided into two intervention groups. Vehicle (PBS) or 50 mg/kg verteporfin was intraperitoneally administered twice a week for 3 weeks. Tumor volume (½ (Length × Width^2^) and weight were measured every other day until sacrifice.

### Immunohistochemistry

The excised tumor tissues were fixed with 4% paraformaldehyde and embedded in paraffin blocks. After cutting and mounting the sections, they were deparaffinized and rehydrated following antigen retrieval. Immunohistochemical staining was performed using anti-Ki-67 (Invitrogen, #MA5-14,520), anti-YAP (Cell Signaling Technology, #14,074), and anti-PD-L1 (Cell Signaling Technology, #13,684). Each marker was detected using the OptiView DAB IHC Detection Kit (Venetana, #760-700). TUNEL assay was performed using the ApopTag^®^ Plus Peroxidase In Situ Apoptosis Kit (#s7101, Millipore). All procedures were performed according to the manufacturer’s instructions.

### Human sample collection and immunohistochemistry

Tumor tissues were obtained from three patients diagnosed with HER2-positive gastric cancer who experienced disease progression following trastuzumab-based therapy at the Seoul National University Hospital. Paired biopsies were collected before and after trastuzumab treatment and post-treatment biopsies were obtained at the time of disease progression. Paraffin-embedded tissue blocks were processed into sliced sections and subjected to antigen retrieval. IHC was performed using antibodies against YAP (Cell Signaling Technology, #14,074) and TEAD (Abcam, #ab97460) and visualization was performed using the OptiView DAB IHC Detection Kit (Venetana, #760-700). H-score analysis utilizing analyzer-assisted interpretation was employed to assess the extent of immunoreactivity.

### Kaplan–Meier analysis

The prognostic significance of YAP1 in patients with GC stratified by HER2 subtype was assessed using the web-based Kaplan–Meier plotter database [27]. Survival data and log-rank p values were extracted from publicly available Gene Expression Omnibus (GEO) datasets, including GSE14210, GSE15459, GSE22377, GSE29272, and GSE51105.

### Statistical analysis

Statistical analyses were performed using SigmaPlot version 10.0 or GraphPad Prism version 8.0. All statistical tests were two-sided unless otherwise indicated in the figure legends. Statistical significance was set at *p* < 0.05.

## Results

### Enhanced ROR2-YAP/TEAD expression in HR cells

**Fig. 1:**
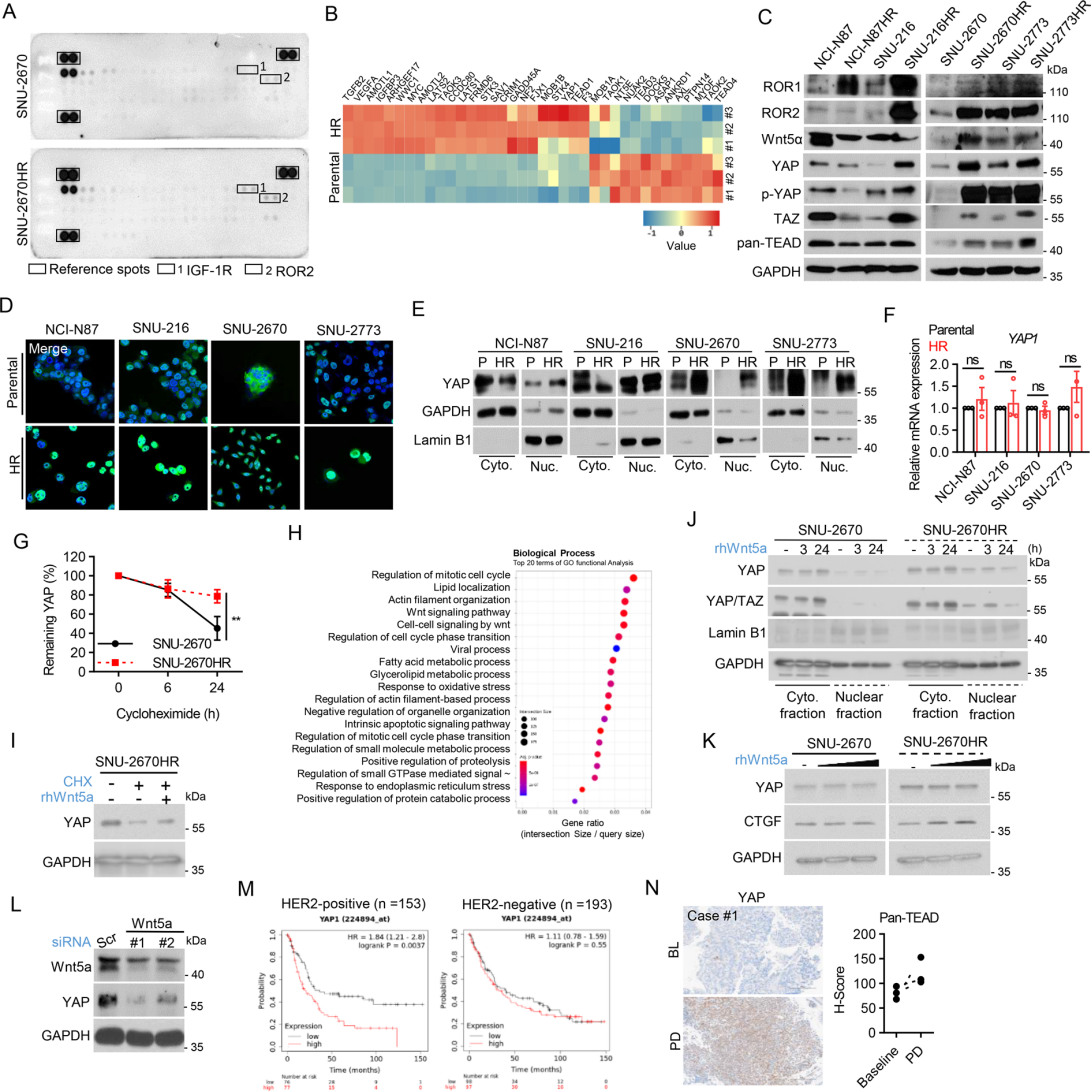
Enhanced ROR2-YAP/TEAD expression in HR cells. **A** Phospho-RTK array of SNU-2670 and SNU-2670HR cells. Array images are displayed with marked rectangles highlighting the two most upregulated genes in SNU-2670HR. **B** Heatmap showing the expression level of a customized gene set representing YAP/TAZ core components and its downstream target genes in SNU-2670 and SNU-2670HR using RNA-Seq. **C** Immunoblot analysis of ROR2-YAP/TEAD pathway components in HR cells. Representative images from two independent experiments are shown. **D** Immunofluorescence analysis of YAP (Green) in HR cells. Nuclei are counterstained with DAPI (Blue). **E** Immunoblot analysis of YAP in the nuclear fraction of HR cells, with Lamin B1 as a nuclear envelope marker. **F** RT-qPCR analysis of YAP expression. Data from three independent experiments are shown as mean ± SEM, ns, not significant. **G** CHX chase assay followed by immunoblot analysis of YAP to determine YAP stability. Cells were treated with 50 µg/ml CHX at the indicated time points and prepared into cell lysates. Relative band intensity of YAP in immunoblot analysis was quantified using ImageJ. Data are shown as mean ± SEM from three independent experiments. Statistical significance was determined by two-way ANOVA: ns, not significant; **p < 0.005. **H** Top 20 enriched GO biological process gene sets in HR cells compared to parental cells, ranked by gene ratio from GO functional analysis. **I** CHX chase assay followed by immunoblot analysis of YAP. Cells were treated with 50 µg/ml CHX alone or in combination with 300 ng/ml rhWnt5a for 24 h and prepared into cell lysates. **J** Immunoblot analysis of YAP following 300 ng/ml rhWnt5a treatment for 3 and 24 h in the nuclear fraction. Lamin B1 was used as a nuclear envelope marker. **K** Immunoblot analysis of YAP and CTGF after cells were treated with 300 ng/ml rhWnt5a for 24 h. **L** Immunoblot analysis of YAP expression in cells after transfection with 50 µM siRNA targeting Wnt5a daily for 2 days. **M** Kaplan–Meier survival curves with log-rank test generated by Kaplan–Meier plotter of patients with GC stratified by YAP1 expression; median cutoff, and HER2 subtypes. HR; hazard ratio. **N** Left panel: Immunohistochemical staining of YAP (20x/ 300 × magnification); right panel: Pan-TEAD H-Score in tumor tissues obtained from patients with HER2-positive GC. *BL* baseline, *PD* progressive disease following trastuzumab-based therapy

To understand the potential mechanisms of resistance to trastuzumab, we conducted proteome profiling via a phospho-RTK array in HER2-positive cancer cells (SNU-2670) and their HR counterparts (SNU-2670HR). Among the 49 tyrosine kinases, ROR2 phosphorylation was elevated in SNU-2670HR, indicating putative dysregulation of downstream pathways (Fig. 1A). ROR2 orchestrates various oncogenic pathways, including the JNK, MAPK, and PI3K/AKT pathways and collaborates with Wnt5a to facilitate YAP nuclear translocation and YAP/TEAD activation (27, 28). Using RNA-Seq, we examined the ROR2-mediated oncogenic signature and identified the transcriptional upregulation of several core YAP/TAZ pathway components, such as LATS1/2 and SAV1, and downstream target genes, such as AMOTL1/2, MYC, and VEGFA, in HR cells through DEG analysis (Fig. 1B). Immunoblot analysis further validated that HR cells, except NCI-N87HR cells, exhibited significant upregulation of YAP, TAZ, TEAD, Wnt5a, and ROR2, indicating robust activation of the YAP/TAZ axis, which was supported by enhanced nuclear YAP expression (Fig. 1C–E).

RT-qPCR confirmed that YAP mRNA expression was comparable between all parental and HR cells (Fig. 1F), consistent with the RNA-Seq findings (HR vs. Parental FC: 1.149287), where the fold change did not meet the threshold for differential expression (RNA FC > 2). However, YAP was elevated post-translationally in HR cells owing to increased protein stability (Fig. 1G). To delve deeper into the mechanism underlying increased YAP stability in HR cells, we conducted GO functional analysis of RNA-Seq data which revealed that Wnt signaling is one of the most enriched gene sets in the HR cells (Fig. 1H).

Exploring the role of Wnt5a in sustaining YAP activation, we found that ectopic treatment with rhWnt5a stabilized YAP and possibly prevented its proteasomal degradation. This led to the enhanced nuclear translocation of YAP, particularly in the HR cells (Fig. 1I, J). ROR2 inhibition with Ozuriftamab abrogated the Wnt5a-mediated stabilization of YAP, suggesting that ROR2 is essential for transducing the Wnt5a signal to promote YAP activation (Fig. S1). Additionally, rhWnt5a upregulated CTGF, a core downstream target of YAP/TEAD, whereas Wnt5a depletion significantly reduced YAP expression, indicating a strong Wnt5a-dependent YAP/TEAD circuit in HR cells (Fig. 1K, L). Verteporfin treatment compromised Wnt5a and ROR2 expression, suggesting the presence of a putative positive-feedback loop for the Wnt5a/ROR2/YAP axis in the HR cells (Fig. S2).

Exploring selected GEO datasets to identify the clinical implications of YAP in patients with HER2-positive GC, a significant correlation between YAP expression and poor prognosis exclusively within HER2-positive GC subtypes, compared to HER2-negatives was observed (Fig. 1M). Immunohistochemical staining of core YAP components in paired tumor biopsies from patients with HER2-positive GC showed elevated YAP expression in one of the three patients during disease progression compared to baseline (Fig. 1N). TEAD expression was also upregulated across all progressive disease samples (Fig. 1N). While these observations were made in a limited sample set, they align with our preclinical findings, supporting a potential tumor-progressive role of the ROR2-YAP/TEAD axis in patients with HER2-positive cancers characterized by trastuzumab resistance.

### HR cells are susceptible to YAP targeting

**Fig. 2:**
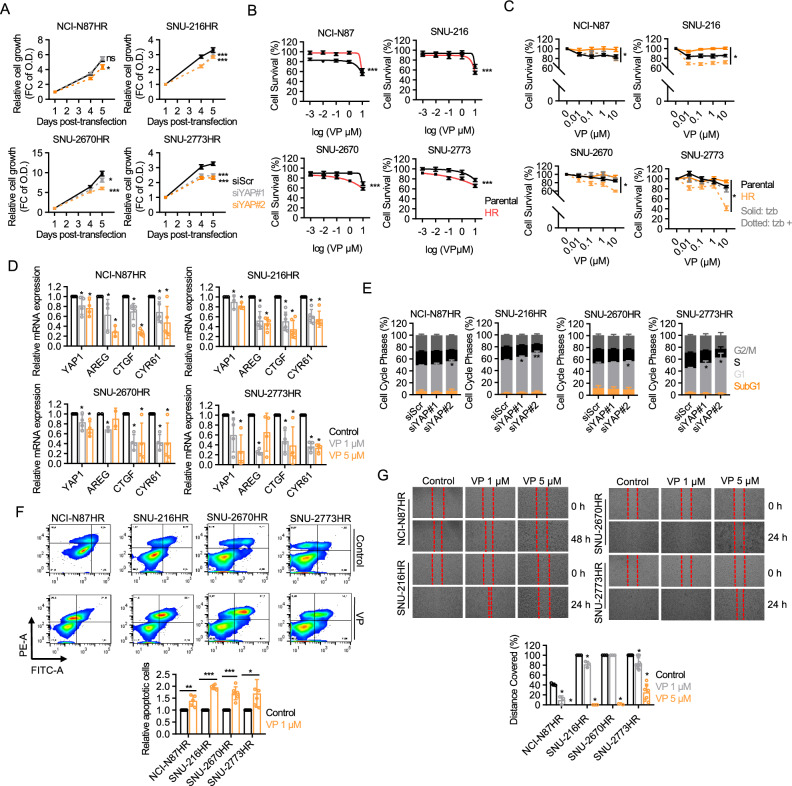
HR cells are susceptible to YAP targeting. **A** Cellular growth rate of HR cells transfected with indicated siRNAs. HR cells were transfected with 50 nM of indicated siRNAs for 48 h and subjected to MTT assays at indicated time points. Data from four independent experiments are shown as mean ± SEM, ns, not significant; *p < 0.05; ***p < 0.001. Statistical significance was determined using one-way ANOVA. **B** Dose–response curves showing the anti-proliferative effect of verteporfin determined by MTT assays following 72 h treatment of verteporfin. Data of at least three biological replicates are shown as mean ± SEM, ***p < 0.001. Statistical significance was determined using a two-way ANOVA. **C** Cell viability analysis determined by MTT assays showing the anti-proliferative effect of trastuzumab in combination with verteporfin (1:1 dose ratio). **D** RT-qPCR analysis of YAP downstream targets in HR cells following 48 h treatment with 1 or 5 µM verteporfin. Data of at least three biological replicates are shown as mean ± SD. **E** Cell cycle analysis following 48 h of incubation after transfection with indicated siRNAs for 48 h. Data of four independent experiments are shown as mean ± SEM, *p < 0.05; **p < 0.005. **F** Upper panel: Representative flow cytometry images from Annexin V apoptosis analysis following 48 h of treatment with 1 μM verteporfin. Lower panel: Bar graphs showing relative apoptotic cells as mean ± SEM, *p < 0.05; **p < 0.005; ***p < 0.001. **G** Upper panel: Representative phase-contrast microscopic images showing cell coverage after treatment with 1 or 5 µM verteporfin at specified time points following wounding. Lower panel: The percentage of migrating cells, quantified using ImageJ, indicating cell movement into the scratched area over time. Data from three experiments are presented as mean ± SEM. Significance is denoted as *p < 0.05

To elucidate the regulatory role of YAP in the oncogenic potential of HR cells, we conducted cell-proliferation assays after YAP was depleted using siRNAs. The results revealed perturbed growth in HR cells upon YAP depletion, except for NCI-N87HR cells, where the expression of the YAP/TAZ/TEAD axis was comparable to that in the parental cells (Fig. 2A). The pharmaceutical inhibition of YAP using verteporfin exerted more potent growth inhibition on YAP-enhanced HR cells than on their parental counterparts (Fig. 2B). Similarly, treatment with CA3, a small molecule known to suppress YAP/TEAD-mediated transcriptional activity, led to preferential growth suppression in YAP-enhanced HR cells, further supporting the YAP-dependent proliferative phenotype of resistant cells (Fig. S3). Moreover, both genetic ablation and pharmaceutical inhibition of YAP improved the therapeutic response of HR cells to trastuzumab, leading to more pronounced anti-proliferative effects exclusively in the HR cells (Fig. 2C, S4). This suggests a fundamental role for YAP activation in trastuzumab resistance. Further analysis revealed that YAP depletion and verteporfin treatment attenuated the transcription of TEAD target genes, such as AREG, CTGF, and CYR61, all of which are associated with diverse tumor-promoting activities, including enhanced metastatic potential and cancer cell proliferation (Fig. 2D, S5) [28]. Moreover, YAP depletion induced cell-cycle arrest in the G1 phase, accompanied by downregulation of cyclin D, cyclin E, and p27 accumulation (Fig. 2E, S6). Verteporfin treatment induced apoptotic cell death in all HR cells, with changes in molecular expression patterns, and significantly reduced their migratory capacity, demonstrating its mechanism of action (Fig. 2F, G, and S7). Thus, these findings indicate the acquired vulnerability of HR cells to YAP inhibition, which may curb the intrinsic oncogenic potential of cancer cells.

### YAP targeting down-regulates PD-L1 expression in HR cells

**Fig. 3:**
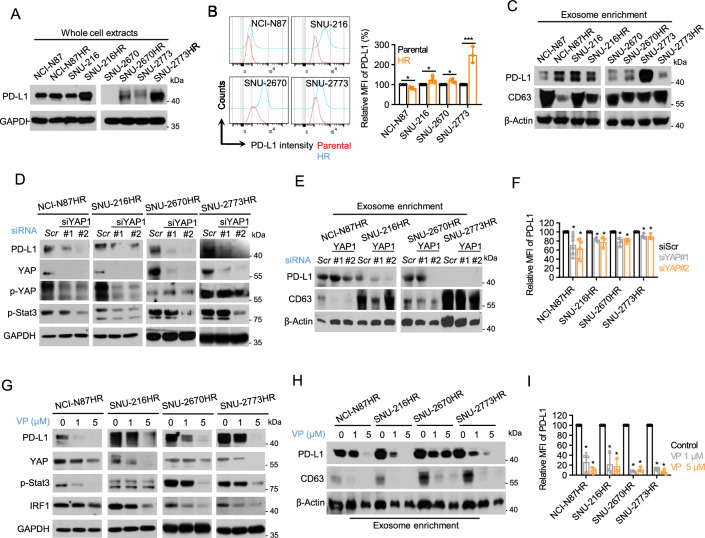
YAP targeting down-regulates PD-L1 expression in HR cells. **A** Immunoblot analysis of PD-L1 in whole-cell lysates (WCLs) from the indicated cells. **B** Flow cytometry assessment of surface PD-L1 levels in the indicated cells. Left panel: representative histograms illustrating relative PD-L1 intensity. Right panel: relative mean fluorescence intensity (MFI) of PD-L1 depicted as mean ± SEM, with significance indicated as *p < 0.05; ***p < 0.001. **C** Immunoblot analysis of PD-L1 in exosomes from the indicated cells, with equal protein loading across all lanes. CD63 served as an exosomal marker. **D** Immunoblot analysis of p-STAT3/PD-L1 in WCLs from cells transfected with specific siRNAs. **E** Immunoblot analysis of PD-L1 in exosomes from cells transfected with specific siRNAs. **F** Flow cytometric analysis of surface PD-L1 levels in indicated cells. Relative MFI of PD-L1 presented as mean ± SEM, with significance denoted as *p < 0.05. **G** Immunoblot analysis of p-STAT3/PD-L1 in WCLs of indicated cells treated with 1 or 5 µM verteporfin for 48 h. **H** Immunoblot analysis of PD-L1 in exosomes of cells treated with 1 or 5 µM verteporfin for 48 h. **I** Flow cytometric analysis of surface PD-L1 levels in indicated cells. Relative MFI of PD-L1 shown as mean ± SEM, with significance indicated as *p < 0.05

The immunoregulatory function of YAP has recently been shown to induce the expression of PD-L1 in a STAT3-dependent manner in various types of tumors [29–31]. Considering that PD-L1 in cancer cells mediates trastuzumab resistance by promoting immune evasion and enhancing cancer cell growth, we focused on the effect of YAP inhibition on PD-L1 expression in HR cells [32, 33]. Total PD-L1 expression consistently increased in the HR cells, as previously reported (Fig. 3A). Moreover, membrane-localized PD-L1 levels were upregulated only in the YAP-enhanced HR cells, compared to their parental cells (Fig. 3B). In comparison, NCI-N87HR cells showed a distinct distribution of PD-L1 localization, with a significant enrichment of exosomal PD-L1, thus suggesting cell line context variability in the resistance mechanism to trastuzumab (Fig. 3C). YAP depletion significantly downregulated the phosphorylation of STAT3 (Tyr 705) and PD-L1 production, resulting in decreased surface retention (Fig. 3D-F). Similarly, YAP inhibition reduced STAT3 phosphorylation and PD-L1 expression in HR cells, with a remarkable reduction in membrane-bound PD-L1 (Fig. 3G, I). In addition, the biogenesis of total exosomes, including exosomal PD-L1, was suppressed by verteporfin, suggesting the potential of YAP targeting to dampen PD-L1-mediated tumor progression and immune evasion of cancer cells (Fig. 3H).

### YAP inhibition in HR cells induces immunogenic cell death and enhances antitumor T-cell response

**Fig. 4:**
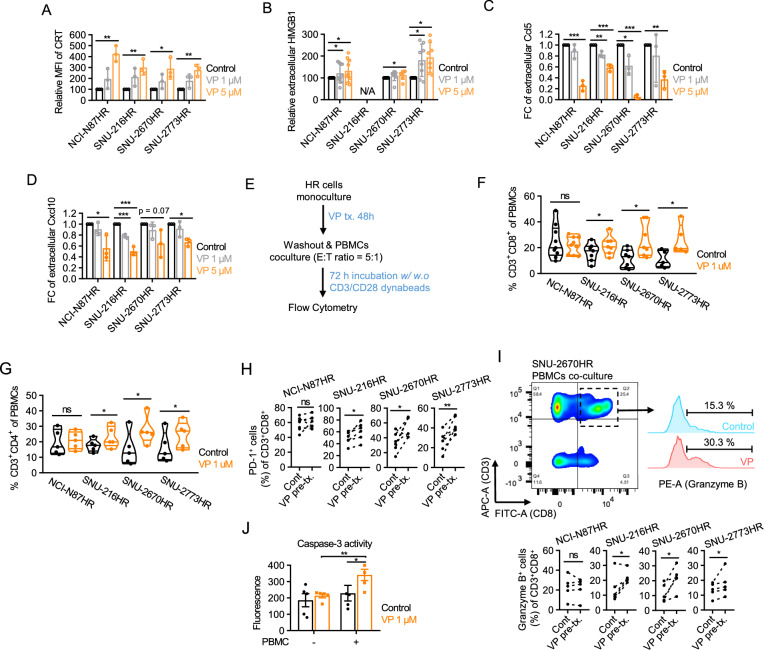
YAP inhibition in HR cells induces immunogenic cell death and enhances antitumor T-cell response. **A** Flow cytometric analysis of cell surface CRT exposure after 48 h treatment with 1 or 5 µM verteporfin. Relative MFI of CRT is shown as mean ± SEM. *p < 0.05; **p < 0.005. **B**–**D** ELISA measurement of extracellular HMGB1 (**B**), Ccl5 (**C**), and Cxcl10 (D) levels following 48 h of 1 or 5 µM verteporfin treatment. Relative protein levels are presented as mean ± SEM. *p < 0.05; **p < 0.005; ***p < 0.001. **E** Schematic of co-culture experiment setup. **F**, **G** Flow cytometric analysis of CD8^+^ (**F**) and CD4^+^ (**G**) T cells in PBMCs stimulated with CD3/CD28 dynabeads after co-culture with HR cells pre-treated with 1 µM verteporfin. Statistical significance was determined using a paired Wilcoxon test. *p < 0.05. **H** Flow cytometric analysis of PD-1^+^ CD8^+^ T cells from co-cultures of PBMCs and verteporfin pre-treated HR cells following CD3/CD28 stimulation. *p < 0.05; **p < 0.005. **I** Flow cytometry analysis of Granzyme B^+^ CD8^+^ T cells from co-cultures of PBMCs and verteporfin pre-treated HR cells without CD3/CD28 stimulation. Upper panel: representative gating plot and histogram showing Granzyme B expression in the CTL population of PBMCs pre-gated from cancer cells by FSC and SSC. Lower panel: statistical significance was determined using a paired Wilcoxon test. *p < 0.05. **J** Caspase-3 activity in HR cells co-cultured with PBMCs. Verteporfin-pretreated SNU-2670HR cells (48 h, 1 µM) were co-cultured with PBMCs for 72 h under CD3/CD28 stimulation. Caspase-3 activity was measured using the EnzChek^®^ Caspase-3 Assay Kit. Fluorescence intensity is shown as mean ± SEM. Statistical significance was determined using a paired Wilcoxon test. *p < 0.05; **p < 0.005

Beyond its role in regulating PD-L1, tumor YAP and YAP in immune cells exert multiple immunosuppressive effects on the tumor microenvironment (TME), allowing tumors to evade immune surveillance [34]. For example, YAP within cancer cells modulates tumor immunity by directly dampening CD8^+^ cytotoxic T cells (CTLs) or indirectly through myeloid-derived suppressor cells (MDSCs) via the paracrine effects of soluble factors or extracellular vesicles [35–37]. These findings highlight that YAP inhibition is an attractive therapeutic strategy for enhancing tumor immunogenicity and antitumor immune responses. To evaluate the potential impact of YAP on the TME of HR tumors, we examined its ability to induce immunogenic cell death (ICD). Verteporfin treatment induced cell surface exposure of calreticulin (CRT) and the extracellular release of HMGB1, both of which are characteristics of ICD-related damage-associated molecular patterns (DAMPs) (Fig. 4A, B). However, YAP depletion concomitantly abrogated the extracellular secretion of the chemokines Ccl5 and Cxcl10, which are key targets of type-I IFNs that promote tumor infiltration and activation of CTLs (Fig. 4C, D). To elucidate the net immunomodulatory effects of YAP inhibition in HR cells, we conducted co-culture experiments with HR cells and PBMCs (Fig. 4E). Verteporfin pretreatment of HR cells increased the numbers of CD4^+^ and CD8^+^ T-cell subsets in PBMCs, indicating their expansion (Fig. 4F, G). The frequency of PD-1^+^ CTLs also increased following YAP inhibition in HR cells, suggesting CTL activation (Fig. 4H). These findings were observed under CD3/CD28 bead stimulation, which provided the co-stimulatory signals required for the ex vivo expansion of PBMCs. Preliminary flow cytometry analysis further revealed that verteporfin-treated HR cells enhanced the activation and frequency of CD14^−^CD11c^+^ conventional dendritic cells (cDCs) within PBMCs, implying a potential role of antigen presenting cells (APCs) in mediating T-cell activation (Fig. S8A, B). Moreover, activation of T cells was observed as an increase in granzyme B^+^ CTLs in PBMCs following co-culture with HR cells treated with verteporfin, even in the absence of external TCR co-stimulatory signals (Fig. 4I). Furthermore, verteporfin pretreatment enhanced caspase-3 activity in HR cells when co-cultured with PBMCs, indicating increased immune-mediated cytotoxicity (Fig. 4J). These findings collectively indicate that YAP inhibition enhances tumor immunogenicity, with its immunostimulatory effects translating into immune-mediated tumor cell killing ex vivo.

### YAP inhibition suppresses the growth of HR tumors in vivo

**Fig. 5:**
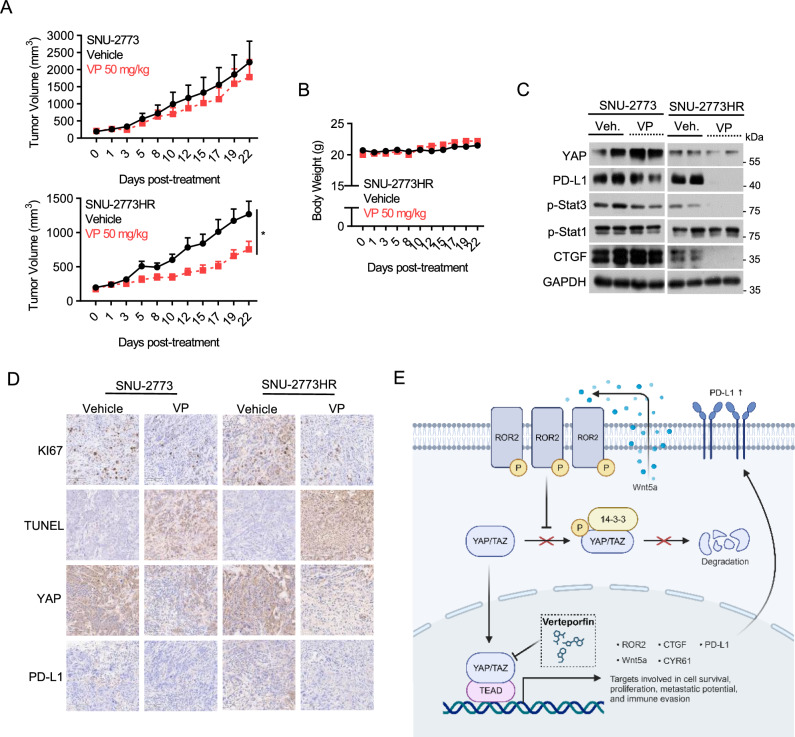
YAP inhibition suppresses the growth of HR tumors in vivo. **A** Growth curves of subcutaneously transplanted SNU-2773 and SNU-2773HR tumors following indicated treatment regimens administered peritoneally twice a week for 3 weeks, Mean ± SEM, ns, not significant; *p < 0.05 (*n* = 5–7 per group). **B** Mean ± SEM data of mice body weight in each treatment arm. **C** Immunoblot analysis depicting YAP/STAT3-PD-L1 axis in tumor lysates obtained at the end of therapy. **D** Representative images of IHC analysis of Ki67, TUNEL, YAP, and PD-L1 in tumors treated with indicated regimens (400 × magnification). **E** Schematic model illustrating YAP pathway involvement in trastuzumab resistance in HER2-positive cancer cells. Enhanced YAP stability via Wnt5a promotes oncogenic YAP signaling, highlighting the therapeutic vulnerability of HR cells to YAP targeting

To assess the antitumor effects of verteporfin on HR tumors in vivo, xenograft tumor models of SNU-2773 and SNU-2773HR were utilized. Verteporfin effectively inhibited the growth of HR tumors without inducing toxicity-related weight loss (Fig. 5A, B). However, verteporfin had no significant effect on the growth of SNU-2773 tumors, indicating the increased susceptibility of HR cells to YAP targeting observed in vitro. In addition, verteporfin treatment resulted in the downregulation of tumor p-STAT3 and PD-L1 expression, along with YAP and CTGF, highlighting the ability of verteporfin to modulate PD-L1 expression in HR tumors (Fig. 5C, D). Furthermore, IHC analysis demonstrated reduced tumor cell proliferation (Ki67) and enhanced apoptotic cell death (TUNEL) in HR tumors following verteporfin treatment, underscoring the enhanced antitumor effects of verteporfin in HR tumors (Fig. 5D).

## Discussion

In this study, we discovered significant upregulation and activation of the YAP/TAZ pathway in trastuzumab-resistant HER2-positive cancer cells, as evidenced by the robust nuclear accumulation and activation of YAP. The ROR2 ligand, Wnt5a, plays a critical role in this process by inducing YAP stabilization, highlighting the profound impact of Wnt5a in HR cells. Clinically, our analysis of patient tumor samples showed that YAP and TEAD expression levels were elevated at the time of disease progression following HER2-targeted therapies in some cases. This observation suggests a potential link to poor prognosis in HER2-positive GC subtypes with high YAP expression. Furthermore, YAP inhibition via verteporfin not only impaired the oncogenic properties of HR cells and sensitized these cells to trastuzumab but also mitigated cancer cell immune evasion by downregulating PD-L1 expression and inducing ICD. In vivo, verteporfin treatment effectively reduced tumor burden in the HR xenograft models, decreased p-STAT3 and PD-L1 expression, and increased apoptosis, underscoring the therapeutic potential of targeting the ROR2-YAP/TEAD axis to overcome trastuzumab resistance.

Wnt5a is the predominant binding partner of FZD receptors and orchestrates non-canonical Wnt pathway activation, including the YAP/TAZ pathway [38]. However, Wnt5a has also been reported to play an opposing tumor-suppressive role by abrogating the YAP/TAZ pathway, as evidenced by its negative feedback in the ROR2-YAP/TAZ pathway in a prostate cancer model [39]. This finding suggests that the pleiotropic role of Wnt5a in the Hippo pathway varies in a context-dependent manner. Therefore, further investigation is required to clarify how Wnt5a regulates YAP activity, including whether this occurs through canonical Hippo components or alternative non-canonical pathways.

In our study, Wnt5a-induced YAP stabilization was not accompanied by any change in phospho-LATS1 levels, suggesting that suppression of LATS1 kinase activity is not involved (Fig. S9). Notably, total LATS1 expression was unexpectedly elevated following rhWnt5a treatment, which we interpret as a potential feedback response to increased YAP activity rather than a direct mechanism contributing to YAP stabilization. We further examined non-canonical regulators such as Src–FAK signaling [40, 41]; however, pharmacological inhibition of Src failed to suppress nuclear YAP localization in HR cells (Figs. S10 and 11). Future studies should explore other candidate pathways and identify key effectors that mediate Wnt5a-induced YAP stabilization. In addition, investigation of how Wnt5a may modulate proteasomal degradation of YAP—including its effects on E3 ligase interactions and ubiquitination—will be necessary to comprehensively delineate the upstream regulation of YAP stability in trastuzumab-resistant cancers. In particular, the role of β-TrCP, a well-known E3 ubiquitin ligase responsible for YAP degradation, warrants further investigation, especially in the context of its regulation by Wnt signaling [42].

With respect to IHC analysis of the tumor tissues from patients with HER2-positive gastric cancer, our findings of elevated YAP and TEAD expression in tissues collected during disease progression following HER2-targeted therapies were derived from a limited number of paired biopsies. While the small sample size restricts the generalizability of our conclusions, our findings suggest an important mechanistic link between the ROR2-YAP/TEAD axis and trastuzumab resistance. Notably, our findings indicate that pharmacological inhibition of canonical trastuzumab resistance pathways—including Src, PI3K/AKT, JAK–STAT, and MEK/ERK—did not suppress YAP expression or prevent its nuclear localization in HR cells (Fig. S10–S12). These data suggest that YAP activation may occur independently of these conventional pathways and highlight the potential of YAP inhibition as a mechanistically distinct and mutually exclusive therapeutic strategy for overcoming trastuzumab resistance. This underscores the necessity of further validation in larger cohorts to establish the clinical significance of this pathway. Future investigations should focus on expanding the sample size, incorporating functional analyses, and evaluating the prognostic, and therapeutic value of ROR2-YAP/TEAD signaling in trastuzumab-resistant HER2-positive cancers.

To address the immunomodulatory effects of verteporfin treatment, we observed the downregulation of MHC-I (data not shown) and type-I IFNs, indicating reduced immunogenicity in HR cells, likely due to the dampened STAT3–IRF1 axis (Fig. 4D, G). Concurrently, verteporfin treatment induced ICD markers, such as calreticulin exposure and HMGB1 release, suggesting enhanced immunogenicity. This duality implies that YAP inhibition may simultaneously attenuate and promote immune responses within the TME. In our co-culture experiments, verteporfin-treated HR cells led to T-cell expansion and activation ex vivo, indicating the immunostimulatory effects of YAP targeting. However, the HLA compatibility between PBMCs and HR cells was not verified in our co-culture system, making it unlikely that the observed T-cell activation resulted from direct antigen presentation by tumor cells. Instead, we propose that DAMPs released from verteporfin-treated HR cells may indirectly stimulate T cells by activating APCs within PBMCs. This interpretation is supported by our preliminary data suggesting enhanced dendritic cell activation following co-culture with verteporfin-treated cancer cells (Fig. S8B). Nevertheless, whether the observed APC activation is directly attributable to ICD-induced DAMPs or other cancer-derived factors remains unclear. Our current data do not establish a causal relationship between DAMP release and APC activation, and further mechanistic studies are required to clarify this potential link. Moreover, these observations are based on limited biological replicates and lack functional validation. The specific immune cell types involved and the underlying mechanisms by which verteporfin modulates T-cell activity have yet to be elucidated. Future studies should aim to incorporate comprehensive phenotypic and functional analyses of APC and T-cell subsets to better define the immunological consequences of YAP inhibition. However, verteporfin treatment led to T-cell expansion and activation ex vivo, suggesting the immunostimulatory effects of YAP targeting.

To comprehensively understand the immunological responses of HR cells to verteporfin treatment in the context of trastuzumab resistance, it is essential to employ models that better recapitulate an intact TME, as the immunodeficiency of xenograft models limits the assessment of tumor-immune interactions. While syngeneic mouse models transduced to express human HER2 are commonly used for HER2-targeted therapy studies, they fail to fully reflect HER2 dependency, reducing their relevance for trastuzumab resistance. Although HER2-dependent models, such as Ba/F3 cells engineered to express human HER2, ensure HER2-driven tumor growth, they originate from pro-B cells rather than epithelial-derived tumors. Despite their ability to form tumors in vivo, they do not recapitulate the characteristics of solid tumors, making them suboptimal for studying trastuzumab resistance in GC and BTC. A promising alternative is humanized patient-derived xenograft (PDX) models, in which tumor cells and immune components originate from the same patient. By infusing PDX-bearing mice with autologous PBMCs, these models better capture patient-specific tumor-immune dynamics. Despite their transient immune engagement and incomplete TME restoration, PDX-based humanized models infused with autologous PBMCs remain the most physiologically relevant system for studying tumor–immune interactions in trastuzumab-resistant HER2-positive cancers. Future studies should focus on optimizing these models to enhance their translational relevance and provide deeper insights into the immunomodulatory effects of YAP targeting.

## Conclusions

Our study highlights the significant upregulation and activation of the YAP/TAZ pathway in HR HER2-positive cancer cells and identifies Wnt5a as a key regulator of YAP stabilization. YAP inhibition via verteporfin shows potential in reducing oncogenic properties and overcoming immune evasion in HR cells, providing a strong rationale for further exploration of the ROR2-YAP/TEAD axis as a therapeutic target for HR HER2-positive cancers. Tumoral IHC analysis of YAP could serve as a potential biomarker and companion diagnostic tool for optimal patient selection among patients with HR tumors. Future research should focus on larger patient cohorts and detailed mechanistic studies to validate and expand these findings and ultimately improve therapeutic strategies for patients with HER2-positive gastric cancers to overcome trastuzumab resistance.

## Supplementary Information

Below is the link to the electronic supplementary material.

Supplementary file1 (DOCX 1043 KB)

## Data Availability

The RNA-Seq data from this study are accessible through NCBI GEO under accession number GSE278175.

## Abbreviations

ADC: Antibody–drug conjugate
YAP: Yes-associated protein
TEAD: TEA domain transcription factor
ROR2: Receptor tyrosine kinase like orphan receptor 2
Wnt5a: Wnt family member 5A
DEG: Differentially expressed gene
PBMC: Peripheral blood mononuclear cell
GEO: Gene expression omnibus
TAZ: Tafazzin
LATS1/2: Large tumor-suppressor kinase ½
SAV1: Salvador family WW domain-containing protein 1
AMOTL1/2: Angiomotin like ½
MYC: MYC proto-oncogene, bHLH transcription factor
VEGFA: Vascular endothelial growth factor A
AREG: Amphiregulin
CTGF: Connective tissue-growth factor
CYR61: Cysteine rich angiogenic inducer 61
PD-L1: Programmed cell death ligand 1
TME: Tumor microenvironment
ICD: Immunogenic cell death
CRT: Calreticulin
HMGB1: High mobility group box 1
DAMP: Damage-associated molecular pattern
CTL: Cytotoxic T lymphocyte
Ccl5: C–C motif chemokine ligand 5
Cxcl10: C-X-C motif chemokine ligand 10
STAT3: Signal transducer and activator of transcription 3
Ki-67: Marker of proliferation Ki-67
TUNEL: Terminal deoxynucleotidyl transferase dUTP nick end labeling
FZD: Frizzled class receptor
CHX: Cycloheximide
IRF1: Interferon regulatory factor 1
