# Supplementary material for: YAP as a therapeutic target to reverse trastuzumab resistance
Source: Gastric Cancer. 2025 Jun 20;28(5):799–813. doi: 10.1007/s10120-025-01630-w (PMC12379669; doi:10.1007/s10120-025-01630-w)
Supplement: Supplementary file 1 — Supplementary file1 (DOCX 1043 KB) [file 10120_2025_1630_MOESM1_ESM.docx]

**Supplementary Information for**

**YAP as a therapeutic target to reverse trastuzumab resistance**

Ah-Rong Nam*_1_, Kyoung-Seok Oh*_1,_ Ju-Hee Bang_1_, Yoojin Jeong_1_, Sea Young Choo_1_, Hyo Jung Kim_1_, Su In Lee_1_, Jae-Min Kim_1_, _2_, Jeesun Yoon_3_, Tae-Yong Kim_1_, _3_, and Do-Youn Oh_1_, _2_, _3_*

_1_Cancer Research Institute, Seoul National University College of Medicine, Seoul 03080, Korea

_2_Integrated Major in Innovative Medical Science, Seoul National University Graduate School, Seoul 03080, Korea

_3_Department of Internal Medicine, Seoul National University Hospital, Seoul 03080, Korea

*These authors contributed equally to this work.

Correspondence:

Do-Youn Oh, MD, Ph.D.

Professor,

Department of Internal Medicine, Seoul National University Hospital, Seoul, Korea

Cancer Research Institute, Seoul National University College of Medicine, Seoul, Korea

101 Daehak-ro, Jongno-gu, Seoul 03080, Korea;

Tel: +82-2-2072-0701; Fax: +82-2-762-9662;

Email: [ohdoyoun@snu.ac.kr](mailto:ohdoyoun@snu.ac.kr)

**
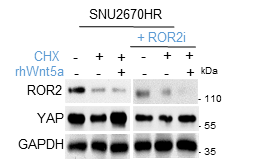
**

**Fig. S1. ROR2 inhibition abrogates Wnt5a-mediated stabilization of YAP.** CHX chase assay followed by immunoblot analysis of YAP. Cells were pretreated with 5 µg/ml Ozuriftamab for 48 h, washed out, and subsequently treated with 50 µg/ml CHX alone or in combination with 300 ng/ml rhWnt5a for 24 h before lysis for immunoblotting.

**
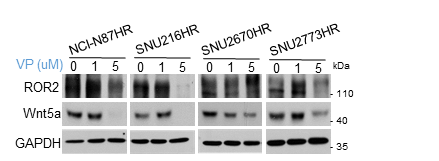
**

**Fig. S2. Verteporfin downregulates ROR2 and Wnt5a in HR cells.** Immunoblot analysis of Wnt5a and ROR2 in cells treated with 1, 5 μM VP for 48 h.


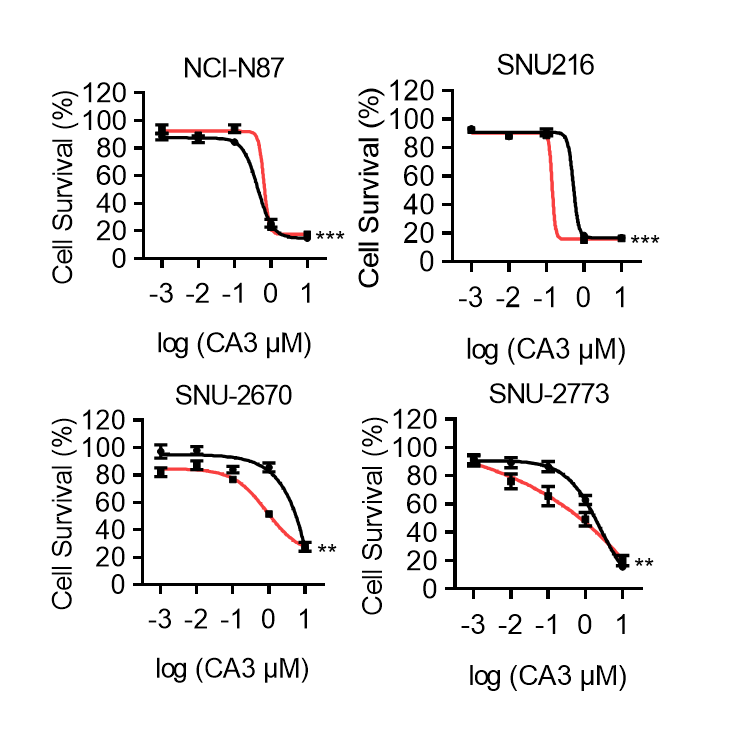


**Fig S3. CA3 selectively reduced the viability of YAP-enhanced HR cells compared to their parental counterparts.** Cells were treated with the indicated concentrations of CA3 for 72 h, and cell viability was assessed using MTT assays. Data from at least three biological replicates are shown as mean ± SEM, **, p < 0.005, ***, p < 0.001.

**
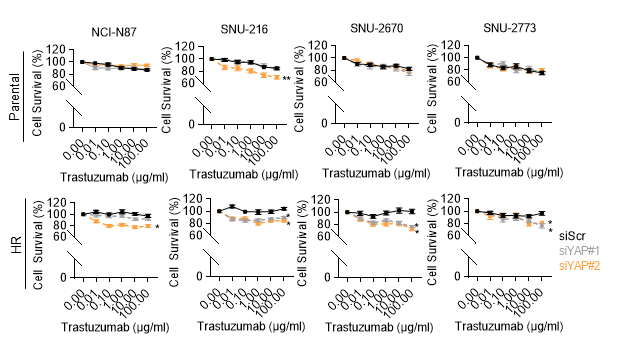
**

**Fig. S4. YAP depletion sensitizes HR cells to trastuzumab.** Cell viability was assessed using MTT assays. Cells transfected with 50 nM siRNAs for 24 h were treated with trastuzumab for an additional 120 h before performing MTT assays. Data from at least three independent experiments are presented as mean ± SEM. *p < 0.05.

**
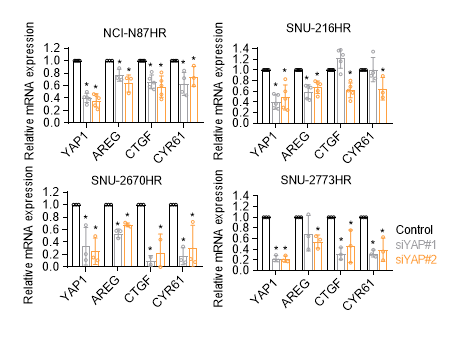
**

**Fig. S5. YAP depletion abrogates downstream transcription.** RT-qPCR analysis of core YAP downstream targets in HR cells following 48 h incubation after transfection with indicated siRNAs. Data of at least three biological replicates are shown as mean ± SD, * p < 0.05.

**
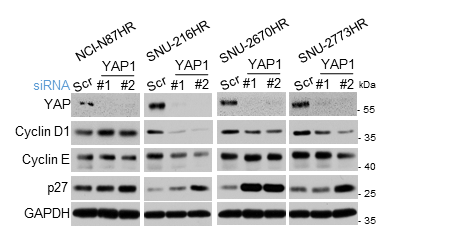
**

**Fig. S6. YAP depletion induces G1 arrest markers in HR cells.** Immunoblot analysis of G1 arrest markers in HR cells after transfection with indicated siRNAs for 24 h.

**
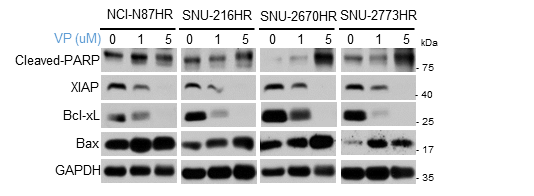
**

**Fig. S7. YAP inhibition induces pro-apoptotic gene expression in HR cells.** Immunoblot analysis of apoptosis-related molecules in HR cells treated with 1 or 5 μM VP for 48 h.

**
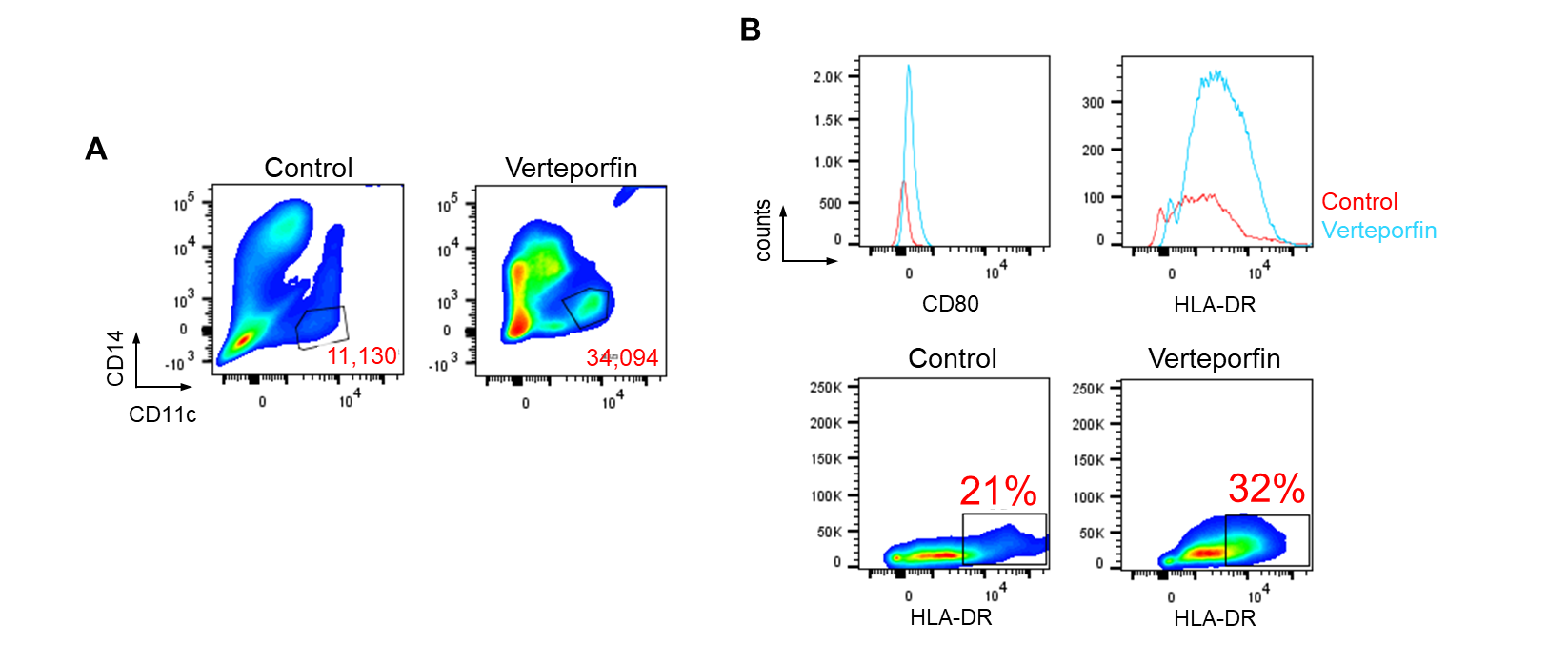
** **Fig. S8. YAP inhibition in HR cells induces the expansion and activation of conventional dendritic cells (cDCs) within PBMCs.** Flow cytometry analysis of CD14⁻CD11c⁺ cDCs following 48-hour coculture of PBMCs with verteporfin-pretreated SNU-2670HR cells under CD3/CD28 stimulation (E:T ratio = 5:1). (A) Absolute counts of cDCs within gated PBMCs. (B) Upper: Histograms showing CD80 and HLA-DR expression in cDCs. Lower: Scatter plot of HLA-DR⁺ cDCs. Data are representative of two independent biological replicates.


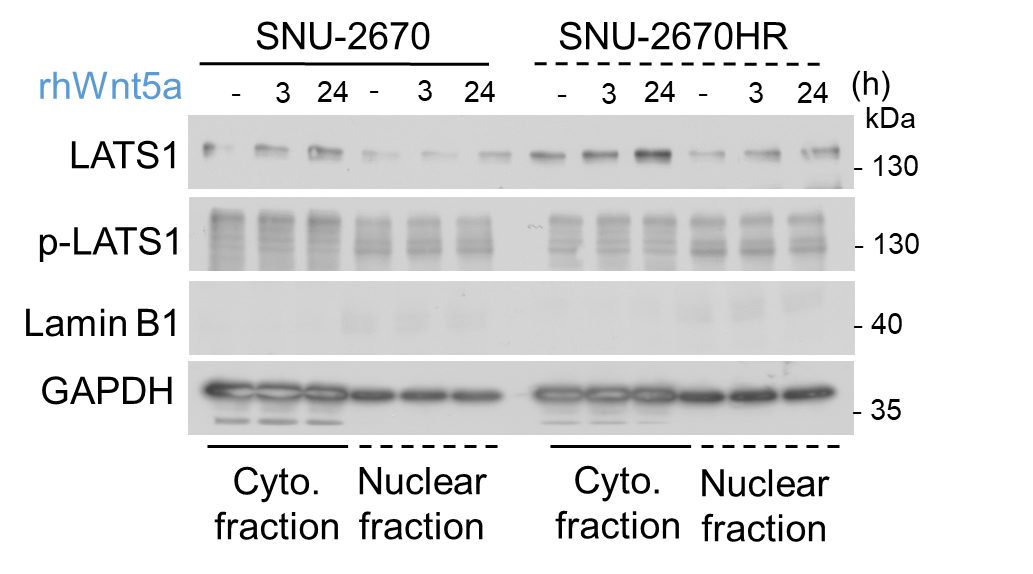


**Fig. S9. rhWnt5a does not affect LATS1 phosphorylation in HR cells.** Immunoblot analysis of YAP following 300 ng/ml rhWnt5a treatment for 3 and 24 h in the nuclear fraction. Lamin B1 was used as a nuclear envelope marker.


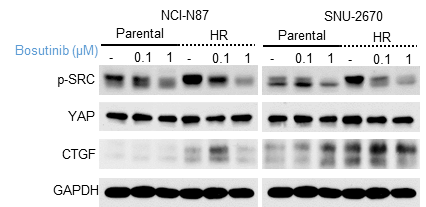


**Fig. S10. Src inhibition does not attenuate the YAP pathway in HR cells.** Immunoblot analysis of total YAP and CTGF in HR cells treated with the indicated concentrations of bosutinib for 48 h.


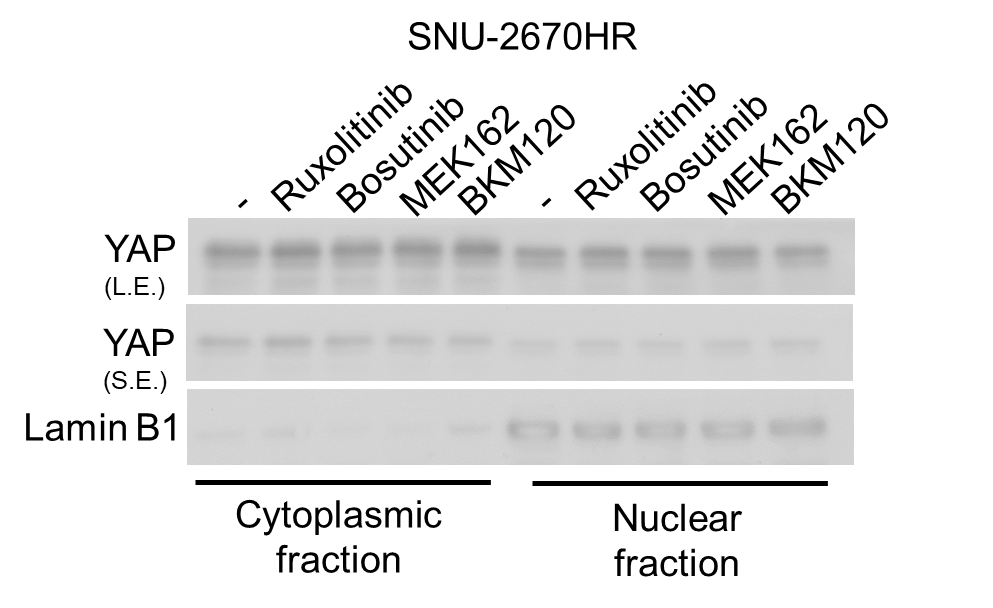


**Fig. S11. Pharmacological inhibition of oncogenic signaling pathways implicated in trastuzumab resistance does not alter YAP nuclear localization in HR cells.** Immunoblot analysis of YAP in the nuclear fraction of SNU-2670HR cells following treatment with 1 μM of the indicated drugs for 48 h. Lamin B1 was used as a nuclear envelope marker.


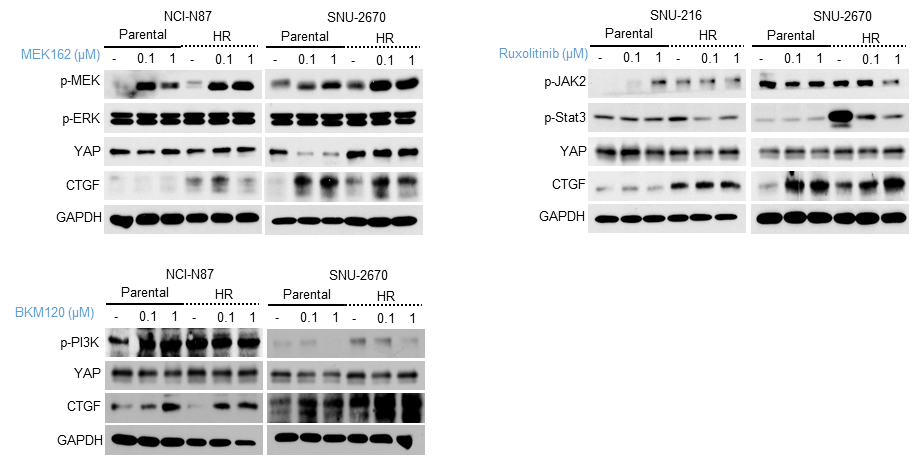


**Fig. S12. Pharmacological inhibition of oncogenic signaling pathways implicated in trastuzumab resistance does no t attenuate YAP signaling in HR cells.** Immunoblot analysis of total YAP and CTGF in HR cells treated with the indicated concentrations (0.1 or 1 μM) of ruxolitinib, MEK162, or BKM120 for 48 h. GAPDH was used as a loading control.
